# Fournier’s Gangrene Diagnosis and Treatment: A Systematic Review

**DOI:** 10.7759/cureus.18948

**Published:** 2021-10-21

**Authors:** Gregory D Lewis, Maliha Majeed, Catherine A Olang, Arjun Patel, Vasavi Rakesh Gorantla, Nelson Davis, Sarah Gluschitz

**Affiliations:** 1 Department of Anatomical Sciences, St. George’s University School of Medicine, St. George’s, GRD

**Keywords:** diagnosis, antibiotics, tachycardia, necrotizing, fournier’s gangrene

## Abstract

Fournier’s gangrene (FG) is a perineal and abdominal necrotizing infection. It is most commonly found in middle-aged men with comorbidities such as diabetes mellitus. Initial symptoms are often indistinct and can rapidly progress to overwhelming infections with a relatively high mortality rate. It is crucial to make a prompt diagnosis so that the patient receives appropriate treatment. Given the importance of the identification of FG, we explored what were the most common signs and symptoms associated with FG, as well as distinguished the gold standard treatment. This systematic review utilized articles identified exclusively through PubMed using key terms such as Fournier’s gangrene, signs, symptoms, and treatment. A total of 37 studies, including a total of 3,224 patients (3,093 males and 131 females), fit our inclusion parameters for relevance that included either the most identifiable presentation of FG or the most effective treatment. From our search, the most common clinical presentation was scrotal and labial pain, fever, abscesses, crepitus, erythema, and cellulitis. Diagnosis is made from clinical findings in conjunction with imaging. The gold standard for treatment was found to be a combination of surgical debridement, broad-spectrum antibiotics, and the administration of intravenous fluids. Further, patient survival was found to be directly related to the time from diagnosis to treatment when they underwent surgical debridement. The importance of early identification for improved outcomes or survival highlights the need for further studies or measures to enhance the identification of the signs and symptoms of FG.

## Introduction and background

In the United States, Fournier’s gangrene (FG) is a rare and fatal form of necrotizing fasciitis, with an incidence rate of approximately 1.6 per 100,000 males [1]. Even with aggressive treatment, the current mortality rate for FG is approximately 40% [2], with literature estimates ranging from 20% to 80% [3]. FG is a rapidly spreading infection that spreads through the superficial and deep fascial layers in the perineal, genital, or perianal regions, causing multiple organ failure and septic shock. Jean Alfred Fournier, a French venereologist, was the first to discover it in 1883 [4,5]. FG is considered to be a polymicrobial infection caused by multiple organisms, including aerobic and anaerobic species such as *Escherichia coli* and *Bacteroides fragilis*. These microbes collaborate to release enzymes that cause tissue necrosis [6]. The bacterial organisms that cause this necrotic infection release collagenases, which cause rapid tissue destruction at a rate of one inch per hour [3], allowing the infection to quickly spread from the genital region to the anterior abdominal wall and vital organs [7].

Even those who survive, suffer from sexual and urological disabilities, with debridement often necessitating multiple reconstructive surgeries [3]. Furthermore, these surgeries frequently necessitate tissue grafting as a means of reconstruction. This is a problem in immunocompromised patients who are unable to accept skin grafts and suffer from poor wound healing [8]. Although FG can affect people of all ages and genders, it is most common in men between the ages of 30 and 60 [9]. Advanced age is a risk factor for FG [2]. FG can develop in patients with no medical history, as well as in those with comorbidities such as diabetes, alcoholism, atherosclerosis, peripheral arterial disease, malnutrition, prostate cancer, human immunodeficiency virus (HIV) infection, leukemia, and liver diseases [10]. Patients with multiple comorbidities are more likely to develop FG and have worse outcomes [2]. The importance of early detection and aggressive treatment in FG recovery cannot be overstated [11].

In this review, our goal was to collect data on the clinical signs and symptoms of FG in the emergency department because early detection is critical to survival. In addition, we examined the most common treatment protocols for initially infected tissue remediation and subsequent assistive rehabilitation procedure.

## Review

Methodology

**Figure 1:**
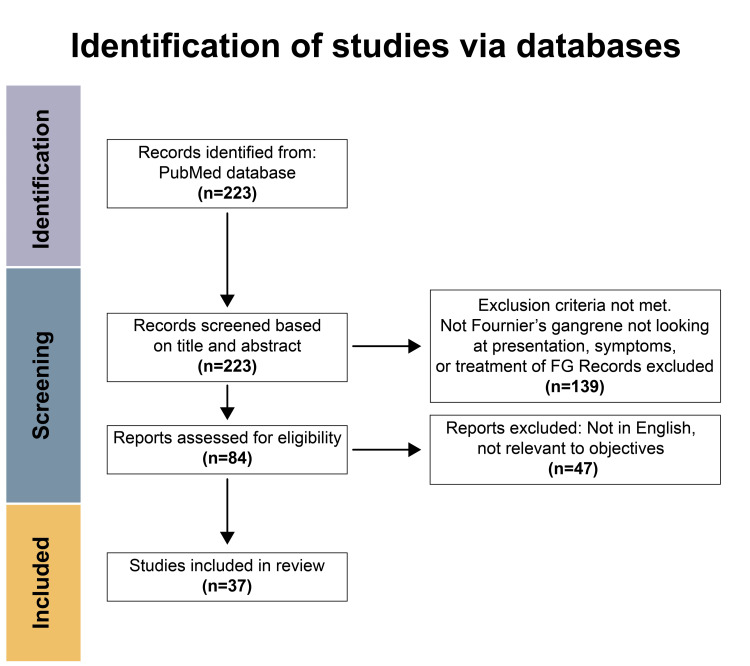
PRISMA flowchart of the literature screening for the presentation, symptoms, or treatment of FG. Screening of the literature was done as described in the PRISMA statement [12]. PRISMA: Preferred Reporting Items for Systematic Reviews and Meta-analyses; FG: Fournier’s gangrene

This review followed the Preferred Reporting Items for Systematic Reviews and Meta-analyses (PRISMA) guidelines [12]. A review of the literature was done in the PubMed database for articles from 2016 to 2021 with the keywords “Fournier’s gangrene” AND (symptom* OR sign OR present* OR identif* OR display OR treatment) on June 12, 2021. Non-English articles irrelevant to FG were not included. Studies investigating other types of necrotizing fasciitis were also excluded. Included studies addressed the signs, symptoms, patient presentation, and identification of FG by the hospital staff. Further, articles that reviewed treatments, prognoses, and outcomes of patients diagnosed with FG were included in this study, as shown in Figure 1 [12]. Studies published in the last five years examining FG were included. We included full-text case studies, systematic reviews, case reviews, literature reviews, retrospective reviews, and original studies. Duplicate studies and books were not included in the review.

Results

Our search yielded 223 articles based on the applied criteria and filters. After screening and assessment of the results based on inclusion criteria and study objectives, 37 articles were included for the presentation, symptoms, or treatment of FG [2,5-7,10-11,13-42]. In these included articles, a total of 3,224 patients were evaluated, including 3,093 males and 131 females. The review indicated that the main symptoms were scrotal and labial pain, fever, abscesses, crepitus, erythema, and cellulitis. The gold standard for treatment was found to be emergent surgical debridement, broad-spectrum antibiotics, and the administration of intravenous (IV) fluids [2,5-7,10-11,13-42].

Associated risk factors

Several modifiable and nonmodifiable risk factors have been linked to FG [2]. Modifiable risk factors are variables that can be changed or adjusted by the patient, either through pharmacotherapy or lifestyle changes. Chronic diseases such as diabetes, substance abuse, and others fall into this category. On the other hand, nonmodifiable risk factors, such as a patient’s age, cannot be changed. In a study of 55 patients with FG, 52.7 % of the patients had pre-existing comorbidities such as diabetes, IV drug use, liver failure, and immune impairment [2,43]. Although the exact mechanism involved in diabetes leading to FG is unknown, it has been suggested that the use of sodium-glucose co-transporter-2 inhibitors may be to blame [44]. Furthermore, due to protein glycosylation and diabetic neuropathy, people with diabetes are more likely to develop lesions. The increased risk of infection in IV drug users is thought to be due to the opportunity for microbial organisms to breach intact skin during needle insertion. Pathogens that would normally be unable to penetrate the skin can be rapidly introduced into deeper tissue via needles, causing various pathogenic processes.

Furthermore, patients with compromised immune systems are less likely to be able to clear bacterial microbes once they have been introduced. Immunosuppressive medications, underlying disease processes such as cancer and HIV, and old age can contribute to an immunocompromised state. Immunosuppressive drugs are used to treat various illnesses, including cancer and autoimmune diseases. They are also administered before organ transplantation.

Evaluation of Fournier’s gangrene

Physical findings: FG has an insidious onset, with 40% of patients presenting with no symptoms, which makes early detection crucial [5]. Pain in the genital or perianal regions, with little to no visible cutaneous damage, is one of the early symptoms [45]. More noticeable features of infection emerge as FG progresses through the deep facial planes. The skin tones of erythematosus patients become dusky and darker. Subcutaneous crepitus with a putrid odor (due to anaerobic microbial activity) may appear toward the end of the infection. Eventually, the infection manifests as gangrene, which has more obvious physical signs [46]. Due to a separate blood supply from the penis and scrotum, the testicles are often spared [47]. In a study, scrotal swelling was the most common symptom in 79% of cases, followed by tachycardia (61%), purulent “dishwater” exudate from the perineal region (60%), crepitus (54%), and fever (41%) [48].

Clinical scoring systems: In a clinical setting, scoring methods are used to determine the likelihood of mortality and to direct physicians to the best treatment options. The Laboratory Risk Indicator for Necrotizing Fasciitis (LRINEC) and the Fournier’s Gangrene Severity Index (FGSI) are two scoring tests that are used. Biomarkers such as serum glucose, C-reactive protein, sodium, potassium, creatinine, heart rate, and body temperature are used in these tests. The LRINEC scale ranges from 0 to 13, with a score of 6 or higher indicating necrotizing soft tissue infections (NSTIs). The FGSI can be used in an emergency to determine the likelihood of survival or death by rating nine clinical parameters on a scale of 0 to 4. In patients with FG, a score of greater than or less than 10.5 indicates a 96% chance of death or survival, respectively [46].

Imaging:To visualize the presence of air and the spread of infection, various imaging techniques can be used. Because 90% of FG patients have subcutaneous emphysema, standard radiography is a quick and valuable tool [5]. Another tool that can be used to make a quick diagnosis is ultrasonography (US). The presence of subcutaneous gas in the perineum and the scrotal area appears as a “dirty” acoustic shadowing on US imaging [48]. The most specific imaging modality for determining the extent of infection is computed tomography (CT), which allows surgical teams to plan debridement accordingly [49]. When other imaging modalities are insufficient to determine the extent of infection, magnetic resonance imaging (MRI) is used [48]. Although MRI can aid in the diagnosis, its utility is limited due to the rapid progression of FG and should not be used to postpone surgical interventions [48].

Treatment

Urgent Surgical Debridement

It is worth noting that successfully managing FG is extremely difficult. This is due to late diagnosis caused by nonspecific symptoms and the rapid progression of necrosis. Hemodynamic stabilization, parenteral broad-spectrum antibiotics, and urgent surgical debridement, in which all necrotic tissue is removed until viable tissue is identified, are the main principles of therapy in FG treatment [50]. According to the findings of a clinical review, it is critical to remove necrotic tissue as soon as possible to prevent infection progression [5]. On the other hand, surgical debridement frequently affects large areas and results in significant deficits. In a retrospective study of 72 patients with FG, a delay in surgical debridement was associated with a significant increase in mortality [51]. Consequently, time and extensive debridement play a large role in a better FG prognosis.

Hyperbaric Oxygen Therapy

Hyperbaric oxygen therapy (HBOT) can be a viable adjunct for a better prognosis in FG treatment [5]. This is based on the pathogenesis of FG; the hypoxia caused by arterial vessel thrombosis leads to ischemia and necrosis, creating a favorable environment for anaerobic bacteria to grow. Therefore, if an environment with optimal oxygen is created, bacterial proliferation slows down. In addition to early surgical debridement, the use of this treatment modality is indicated in patients who are unresponsive to conventional therapies such as sterile honey and maggots. However, it is essential to note that some studies have reported an increased mortality rate in patients receiving HBOT [52]. This can be attributed to the fact that patients with more severe presentations were administered HBOT. Thus, there is a risk of bias. It may be challenging to correlate the two (increased morbidity and HBO therapy) because of the rarity of the disease, its intrinsic complexity, and the limited availability of HBOT chambers.

Negative Pressure Wound Therapy or Vacuum-Assisted Closure

After surgical debridement of all the necrotic tissue, vacuum-assisted closure (VAC) can be used to promote wound healing physiologically [23] while reducing the need for reconstructive surgery with skin grafting in the future [14]. There is also evidence to suggest that it can speed up tissue healing [24]. VAC is based on the negative pressure vacuuming that leads to the increase in blood supply and inflammatory cell migration to the affected area. This leads to granulation tissue formation, as well as the clearance of bacterial contamination, toxins, exudates, and debris [23]. VAC therapy involves applying a sterile open-cell foam sponge to the wound and adding transparent adhesive drapes and a noncollapsible tube, which is connected to a portable pump that provides negative pressure to this air-tight environment of the wound [24]. Because of the clinical benefits of VAC compared to traditional wound dressing, it is now being used more frequently than traditional wound dressing, which requires multiple changes, and, in some cases, requires subsequent surgeries to clear the necrosis. Knowledge of the predisposing and risk factors on the initial presentation can allow performing diverting procedures such as hemodialysis before it is too late.

Discussion

FG, a rapidly progressing, high-mortality condition, is frequently misdiagnosed because of the nonspecific nature of the symptoms. Therefore, it is crucial to identify the pathological process as soon as possible to ensure the best possible recovery. A clinical diagnosis is made using a combination of physical findings, standardized scoring, and imaging, as well as the patient’s risk factors.

Diabetes mellitus is the most common risk factor for FG, which typically manifests in men over the age of 55. Although FG has traditionally been portrayed as a disease primarily affecting men, identification of FG in female patients has improved [2,42].

FG begins with symptoms such as fever and perineum edema and is occasionally accompanied by disproportionate pain in relation to how the tissue appears. Crepitus, purulent discharge, and necrosis become distinct diagnostic features of necrotizing fasciitis as the infection spreads. Scoring with LRINEC and FGSI can be a useful tool in determining survivability in FG patients.

The presence of various biomarkers is scored by assigning a numerical value, with values above a standardized threshold indicating a higher risk of death [42]. Subcutaneous crepitus, a distinguishing feature of anaerobic microbial infections, can be detected using imaging techniques such as radiographs, US, CT, and MRI. Imaging can also help the surgical team determine the extent of the spread [48].

Once the diagnosis has been confirmed, immediate surgical intervention is required. The foundation of all FG treatments is a combination of surgical debridement to remove necrotic tissue and broad-spectrum antibiotic administration [5]. The rate at which a patient receives surgical treatment has a direct correlation with survival. Supplemental HBOT treatment can help stop bacterial growth, but it has also been shown to have a negative impact on disease prognosis if surgical intervention is delayed. After the surgery, recovery from a major procedure presents new challenges and may necessitate multiple wound dressings, skin grafts, and plastic surgery. Negative pressure wound therapy and VAC are two postsurgical treatments that improve wound healing by encouraging new blood vessel growth and immune cell migration [14]. All the studies included in this review are presented in Table 1.

**Table 1:** Studies exploring the signs, symptoms, and treatment of FG. FG: Fournier’s gangrene; FGSI: Fournier’s Gangrene Severity Index; NPWT: negative pressure wound therapy; HBOT: hyperbaric oxygen therapy; VAC: vacuum-assisted closure; ACCI: age-adjusted Charlson Comorbidity Index; HBO: hyperbaric oxygen

	Author(s)	Country	Study population	Signs and symptoms	Treatment	Conclusion
1.	Singh et al., 2016 [5]	United Kingdom	Case review; 1,726 cases; males	Scrotal pain, swelling, erythema, systemic fever, rigor, and tachycardia	Broad-spectrum antibiotics, surgical debridement, with, on average, 3.5 surgical debridement per patient, and HBOT	FG is a surgical emergency and urgent, complete debridement is the foundation of patient survival
2.	Chernyadyev et al., 2018 [42]	Russia	Literature review; 7 males	Fever, ulceration in the balanus, prepuce, skin of the penis, or scrotum	Emergency surgical intervention in combination with antibacterial and detoxification therapy	The doctor should be able to differentiate venereal diseases from the initial stages of FG to prevent the development of the disease
3.	Kuzaka et al., 2018 [10]	Poland	13 male patients; median age of 59.6	Necrosis of the tissues in the affected areas (predominantly in the genital region), intense pain, and tenderness	All patients underwent surgical resection of all necrotic tissues. Prior to surgery, all patients underwent intensive intravenous fluid replacement and were treated with broad-spectrum triple antimicrobial therapy	Favorable outcome of FG can be achieved with rapid diagnosis, urgent surgical debridement of all necrotic tissues, and broad-spectrum antibiotics against aerobic and anaerobic bacteria
4.	Louro et al., 2019 [13]	Portugal	14 males, 1 female; mean age of 66.9	Two patients had perianal and another two had skin abscesses. Eight (53.3%) patients had no identifiable source of FG	Early recognition and extensive necrotic tissue debridement, along with prompt and adequate antimicrobial treatment, are the mainstay of FG management	FG is a rapidly progressing necrotizing fasciitis of the perineum arising from urologic, colorectal, or skin foci, and must be considered as a surgical emergency
5.	Hong et al., 2017 [14]	Korea	18 males, 2 females; mean age of 61·8 ± 12·7	Perineal pain (60%), perineal edema (50%), and anal bleeding (55%). Perineal necrosis or discharge (10%), difficulty in defecation (5%), difficulty in voiding (10%), suprapubic pain (5%), fever (10%), and general weakness (25%)	Of the 15 patients admitted to an intensive care unit, 11 underwent colostomy, and four required skin grafts for wound healing. Two patients required VAC dressing without additional surgery	FG is uncommon and doctors’ experience is severely limited. It is difficult to diagnose FG before necrosis or gangrene sets in
6.	Lauerman et al., 2017 [15]	United States	Retrospective review; 168 patients	Patients with FG often present with concurrent medical comorbidities, sepsis, and organ dysfunction	Debridement and antibiotics. A total of 28 patients received 7 days or less of antibiotic therapy, 52 patients received 8–10 days of antibiotic therapy, 56 patients received 11–14 days of antibiotic therapy, and 32 patients received 15 days or more of antibiotic therapy	Shorter antibiotic courses and initial antibiotic selection exclusive of many resistant organisms were not associated with worse outcomes in FG
7.	Dos-Santos et al., 2018 [16]	Brazil	40 patients; 29 (72.5%) males, 11 (27.5%) females; mean age of 51.7 ± 16.3 years	All patients had clinical signs such as pain, bulging, and erythema. The majority (30 patients, 75%) had perianal abscess as the probable etiology	Antibiotic therapy and surgical treatment, with a mean of 1.8 surgeries per patient	These patients had long hospitalization and high mortality. Data suggest the need for improvements in the emergency services, early diagnosis and treatment of the disease, and reducing its morbidity and mortality
8.	Çalışkan et al., 2019 [17]	Turkey	36 FG cases; 35 males, one female	The most common symptom was swelling. The most common predisposing factor was diabetes mellitus	Broad-spectrum antibiotics and urgent surgery. After urgent surgical debridement, daily dressing with nitrofurazone was done	FG is a life-threatening urological emergency with a high mortality rate
9.	Sparenborg et al., 2019 [18]	United States	42 patients; mean age was 53.45; male/female ratio was 39:1	Scrotal cellulitis, scrotal abscess, perirectal/perianal abscess, persistent urethral catheterization, hidradenitis, infected Bartholin cyst, and decubitus ulceration	An average of 3.2 surgical procedures	The FGSI score predicts a greater likelihood of more surgical interventions, longer hospitalization, sepsis, complications, and mortality
10.	Fonseca-Muñoz et al., 2020 [19]	Mexico	Two male patients	Perianal pain, redness, fever, perianal edema, a central ulcer draining fetid, purulent material, and necrosis of the scrotum	One surgical debridement with antibiotics, followed by addition of maggots	Only one surgical debridement was required when maggots were used (normal is about three)
11.	Ferretti et al., 2017 [11]	United States	20 patients; 19 males, 1 female	The scrotum was the primary site of involvement in 12/20 patients. The perineum was the primary site in 8/20 and penis the primary site in 1/20 patients. 50% of the patients had abdominal wall involvement and 25% had documented rectal involvement	Broad-spectrum intravenous antibiotics and immediate surgical debridement. In recent decades, NPWT and HBOT were introduced as adjunctive treatment modalities	Standard protocol of broad-spectrum antibiotics, initiation of sepsis protocol, and prompt surgical debridement in FG are associated with a 15% mortality rate. These patients had an average length of stay of 32 days
12.	Sockkalingam et al., 2018 [20]	India	34 patients; mean age of 50 ± 11.13; male-to-female ratio of 33:1	Scrotal pain, rapidly spreading cellulitis and erythema, fever, and features of systemic toxicity	Intravenous fluids, third-generation cephalosporins, aminoglycosides and metronidazole, surgical wound debridement, and reconstructive procedure were performed	The mortality can be minimal with aggressive medical and surgical management
13.	Beecroft et al., 2021 [21]	United States	143 patients; 110 males, 33 females	A total of 11 patients with vulvar abscess, four with perirectal or gluteal abscess, four with perineal abscess or cellulitis, four with thigh abscess, three with a history of cancer of pelvic origin, one with prolonged immobilization, and one postprocedural	Surgical debridement is essential in the management of FG	Despite the higher relative risk of FG in men, females share many of the same presenting factors as males
14.	Yücel et al., 2017 [22]	Turkey	25 patients; 11 males, 14 females	Pain, inflammation, edema, necrosis, and subcutaneous crepitation are often noted in the involved region	Effective resuscitation, wide-spectrum antibiotherapy, and aggressive debridement of necrotic tissues form the foundation of successful therapy	VAC provides efficient wound care, reduces edema, augments blood flow, and hastens tissue healing
15.	Ozkan, 2016 [23]	Turkey	12 patients; 7 males, 5 females, (mean age: 62·4)	Fever, perianal pain, anal pain, necrosis, scrotal swelling, and perianal necrosis	Urgent resuscitation with fluid, broad-spectrum parenteral antibiotics, and blood transfusions, if needed. All patients underwent immediate extensive surgical debridement with resection of all necrotic tissue until viable tissue was identified	The combined use of NPWT and Flexi‐Seal shows promising results in wound management
16.	Ioannidis et al., 2017 [24]	Greece	24 patients; mean age of 58.9 years; 20 males (83.4%), 4 females (16.6%)	Ulceration of the epidermis, presence of neutrophilic exudate, thrombosed vessels, and necrosis and abscessation of the subcutaneous fat tissue. Comorbidities were present in almost all patients	Extensive surgical debridement and broad-spectrum antibiotics until microbiological culture results were received	Early diagnosis, aggressive thorough surgical treatment, and administration of proper antibiotic treatment lead to better outcomes
17.	Garg et al., 2019 [25]	India	72 patients; all males; mean age of 56.27 ± 19.27	Perineal discomfort (n = 62; 86.1%) and fever (n = 48; 66.7%)	Multidisciplinary approach, including parenteral antibiotics and urgent surgical debridement	Advanced age, diabetes mellitus, renal impairment, leukocytosis, altered sensorium, shock at presentation, and high FGSI and ACCI scores can be used as predictors for poor response and prognosis
18.	Joury et al., 2019 [2]	United States	Case study, a 51–year-old male	Scrotal pain and swelling for a one-week period without preceding trauma to the perineal area	Emergent surgical debridement for extensive necrotizing fasciitis. NPWT and subsequent daily dressing of the extensive wound and antibiotics	Early initiation of antibiotics, surgical intervention, and proper wound care postoperatively are cornerstones of recovery
19.	Semenič and Kolar, 2018 [26]	Slovenia	Case study; a 30-year-old male	Swelling and pain in the gluteal area	Emergency surgery with excessive fasciectomy and neurectomy were performed; broad-spectrum antibiotic therapy was administered	FG is a surgical emergency. Treatment includes broad-spectrum parenteral antibiotic therapy and, most importantly, surgical debridement. Any delay in treatment can dramatically increase mortality
20.	Klement et al., 2019 [27]	Germany	Case study, a 53-year-old male	The occurrence of a sharp pain in the perianal region	Extensive surgical debridement and broad-spectrum antibiotics until microbiological culture results were received	Early diagnosis, aggressive thorough surgical treatment, and administration of antibiotic treatment comprise the cornerstones of the outcome of this disease
21.	El-Shazly et al., 2016 [7]	Egypt	28 patients, males. Group 1 managed with conservative treatment (17 patients), and group 2 underwent urgent exploration (11 patients)	Infection in FG commonly starts as a cellulitis at the sites of entry of infection, depending on the source of the infection, which is commonly the scrotum, perineum, or perianal region	Group 1 managed with conservative treatment, and group 2 managed with urgent exploration with longitudinal hemiscrotal incision starting from the external inguinal ring.	Early exploration and debridement in FG has a better clinical outcome with reduced hospital stay and number of debridement sessions than conservative treatment with delayed debridement
22.	Amin and Blazevski, 2019 [28]	Australia	Case study, a 45-year-old male	An itchy sensation at the dorsum of the distal penile shaft, which had subsequently excoriated and intermittently bled. He had some pain of the external genitalia. Severe deformity of the shaft of the penis, which had been progressively worsening over the course of five days	Intravenous fluid resuscitation, commenced on intravenous vancomycin, meropenem and clindamycin, and was taken to the operating theater for debridement	Penile self-injections performed to attempt to increase penile size have been reported to cause latent pain, ulceration, and FG
23.	Arora et al., 2019 [29]	India	50 patients; all males; mean age of 53 ± 16 years; mortality rate of 24%	Local pain, local swelling, foul smelling discharge, and fever	Surgical debridement. The average number of debridement across all patients was 1.32, ranging from one to three with no significant correlation to mortality	Increasing age, diabetes, alcoholic liver disease, bed-ridden status, and delayed hospital presentation are associated with higher mortality in FG
24.	Morais et al., 2017 [30]	Portugal	Retrospective study; 19 patients; 14 males, 5 females; median age of 70 (62; 78.5); mortality rate of 21%	Necrotizing fasciitis evolving the anterior and/or the posterior perineum	The debridement was aggressive, seeking to remove all contaminated and nonviable tissue. All wounds were washed intraoperatively with H_2_O_2_ and left open, and drainage tubes were placed where there was a subcutaneous tunnel between two incisions.	Increased affected body surface area is a useful prognostic factor in FG
25.	Anheuser et al., 2018 [31]	Germany	62 patients; males; without HBOT (group A, n = 45), and with HBOT (group B, n = 17).	Demographic data showed no significant differences between the group that received HBOT and the group that did not	45 patients were treated with standard therapy: urgent surgical debridement and parenteral broad-spectrum antibiotics (group A, non-HBOT). The other group, with 17 patients, was additionally treated with a hyperbaric procedure (group B, HBOT)	Demographic data showed no significant differences between the group that received HBOT and the group that did not
26.	Lin et al., 2019 [32]	Taiwan	118 male patients	Local tenderness, erythema, swelling, purulent discharge, blister or bullae, gangrene or necrosis, ﬂuctuation or crepitation of the perineal or genital region, and possible systemic toxicity, such as fever, shock, or multiorgan dysfunction	All patients received immediate surgical intervention and intensive care	The overall mortality of 118 FG patients was 14.4%
27.	Eksi et al., 2020 [33]	Germany	80 patients; 65 (81.2 %) males, 15 (18.7 %) females; mean age of 55.1 ± 7.6	75% had increased scrotal volume, 89% had scrotal or genital pain, and 72% had elevated fever during the admission. The mean time between onset of complaints and admission to hospital was 4.6 days, indicating a slight delay	All patients underwent radical debridement for necrotizing tissues within 12 hours after admission to the emergency department. Intravenous antibiotics were maintained postoperatively	With the improvements in FG disease management, mortality rates are decreasing, but long-term hospital stay has become a new problem
28.	Selvi et al., 2019 [34]	Turkey	30 male patients	Diabetes mellitus, hypertension, chronic alcohol use, paraplegia/hemiplegia/bedridden, diverting cystostomy, diverting colostomy	Aggressive surgical debridement of all necrotic tissue within 24 hours of hospital admission, administration of broad-spectrum antibiotics, daily wound dressing, and repeated resections of infected and necrotic tissue	The need for early reconstruction and improved quality of life have gained importance as the survival rates associated with FG have increased
29.	Wetterauer et al., 2018 [6]	Switzerland	20 male patients; median age of 66 (46–73)	Scrotal/perineal swelling, tenderness on palpation, and poorly demarcated erythema, yet no visible necrosis of the skin	Surgical debridement and standard empiric antibiotic regimen. Extensive and sequential debridement regularly resulted in significant tissue defects	FG was associated with a mortality of 15% despite maximum multidisciplinary therapy
30.	Doluoğlu et al., 2016 [35]	Turkey	39 patients; 36 males; 8 (20.5%) died and 31 (79.5%) survived	The diagnosis of FG was based on the presence of fever of >38°C, scrotal or perianal erythema and swelling, purulent malodorous discharge, and fluctuation or crepitation at the wound	An emergent aggressive debridement was performed in all patients. All necrotic tissues were removed until healthy, bleeding tissues were seen. Cystostomy catheters were used, and all patients underwent dual antibiotic treatment	This rapidly progressive disorder is caused by impaired host resistance secondary to reduced cellular immunity, leading to a suppurative bacterial infection
31.	Ghodoussipour et al., 2018 [36]	United States	54 male patients; mean age of 49.3	All patients had an initial wound culture positive for bacterial growth, and 77% had polymicrobial infections identified on culture	Aggressive intravenous fluids and early plastic surgery consultation and multidisciplinary care to reduce morbidity and improve outcomes. Multiple staged debridements were performed on all patients, and daily wound care was performed. Patients were also routinely cultured.	Early emphasis on supportive care, nutrition, and involvement of reconstructive surgeons can decrease the length of stay in patients with FG
32.	Chia and Crum-Cianflone, 2018 [37]	United States	59 cases; mean age of 56; 71% of the patients were males	Groin erythema, presence of a local abscess, scrotal swelling, and altered mentation. Fever was present in only 20% of the patients. Laboratory values included leukocytosis and kidney injury with creatinine of >1.2 mg/dL. 19% of patients had septic shock	Treatment included empiric broad-spectrum antibiotics as well as incision and drainage of abscesses and/or surgical debridement and excision of affected tissues	Despite advancements in medicine, it remains a life-threatening disease that warrants current evaluation of the causative pathogens to optimize treatment outcomes
33.	Pehlivanlı and Aydin, 2019 [38]	Turkey	23 patients; 19 males, 4 females; mean age of 65.91 ± 16.34	Perianal abscess and type 1 diabetes mellitus. Escherichia coli was the pathogen identified most often	In addition to surgical therapy, electrolyte regulation, fluid resuscitation, and broad-spectrum antibiotic agents were administered	FG necessitates urgent and aggressive surgical treatment
34.	Perry et al., 2018 [39]	United States	Retrospective review of 17 patients	Clinical suspicion of FG was based on genital and perineal cellulitis, fever, leukocytosis, and confirmation of tissue necrosis upon surgical exploration	The mean number of total surgeries including simultaneous debridement and reconstruction was 5.5. As an adjunct to reduce bacterial bioburden, antimicrobial irrigations were routinely administered postoperatively	Early surgical exploration and debridement may minimize the total number of surgeries and the hospital length of stay
35.	Lin et al., 2019 [40]	China	60 patients; 56 males, 4 females	Scrotal/labial swelling, scrotal/labial pain, scrotal/labial redness, perineal pain, perineal pruritus, crepitus, and fever	Aggressive surgical debridement of nonviable tissue and triple empirical antibiotics were used in all cases	FG originating from the anorectal region can be rapidly progressive and life-threatening. Infection can spread superiorly to the genital region without the involvement of perineal tissue
36.	Creta et al., 2020 [41]	Italy	161 patients; 94.4% males	FGSI was calculated based on body temperature, heart rate, respiratory rate, hematocrit and leukocyte counts, serum sodium, serum potassium, creatinine, and bicarbonate levels	All patients underwent broad-spectrum intravenous antibiotic therapy empirically upon diagnosis. Therapy was based on culture results. Aggressive debridement was performed in 139 patients. A total of 72 patients underwent HBOT.	HBOT, as an adjunctive treatment in patients with FG, significantly reduces disease-related mortality
37.	Kuchinka et al., 2019 [9]	Poland	4 male patients	Pain, inflammatory edema, presence of blisters filled with serous fluid, crackling. Risk factors include males, diabetes, hypertension, malignant neoplasms, alcoholism, and immunosuppression.	Surgical excision of necrotic tissues, antibiotic support, equation of fluid, electrolytes, and acid–base-balance, level of glycemia	Treatment requires early surgical approach with excision of necrotic tissues, antibiotics support and treatment with HBO in some cases

Limitations of the study

While identification is improving in female patients, they may continue to be under and misdiagnosed as a majority of studies used in this review focus on signs and symptoms in males. Further, while early diagnosis and prompt surgical intervention are more likely to lead to a better outcome, FG continues to have high morbidity and mortality rates, and favorable outcomes are not guaranteed.

## Conclusions

Necrotizing fasciitis, such as FG, is a life-threatening infection and necessitates immediate medical attention. Given the rapid progression of this infection, it is critical for physicians to rapidly identify vulnerable populations at high risk of developing this infection and recognize the clinical presentation to correctly diagnose the patients at an early stage. This systematic review found that the most common clinical presentations were perineal pain, erythema, cellulitis, fever, abscesses, and crepitus. Patients may present with many or only a few symptoms depending on the stage of infection. Our search found a range of treatment options, including HBOT and less conventional therapies such as sterile honey and maggots. The most effective treatment protocol for patient survival was the administration of broad-spectrum antibiotics along with emergency surgical debridement. With clinical training and early recognition, mortality can be reduced in patients with FG. Because the presentation of FG can sometimes be nonspecific and vague, it is important for future research to look for more definitive characteristics that can differentiate FG from similarly presenting conditions.
